# Longitudinal Engagement in Modifiable Lifestyle Behaviors and Dementia Risk

**DOI:** 10.1093/geroni/igaa057.188

**Published:** 2020-12-16

**Authors:** Roger Wong

**Affiliations:** State University of New York Upstate Medical University, St. Louis, Missouri, United States

## Abstract

Dementia is a major public health threat as there are currently no definitive methods to prevent, treat, and cure the disorder. Recent efforts have focused on modifiable lifestyle behaviors to reduce dementia risk. Yet, the majority of these studies have utilized cross-sectional data or are limited to specific geographic areas. The purpose of this study was to explore how longitudinal engagement in modifiable lifestyle behaviors (physical activity, smoking, and social contacts) influence dementia risk. This study analyzed eight annual waves (2011-2018) of prospective data from the National Health and Aging Trends Study, a large nationally representative U.S. sample of older adults. Each wave, physical activity was measured as engagement in vigorous physical activities; smoking was measured as current engagement in cigarette smoking; and social contacts was measured as visiting friends/family outside of their home. The dependent variable was number of years to a new dementia diagnosis. Multivariate analyses were conducted using the Cox proportional hazards model with survey sampling weights applied for a national sample of 6,800 community-dwelling older adults dementia-free at baseline. After controlling for sociodemographics (age, sex, race, etc.) and health (physical health, chronic disease, etc.), longitudinal engagement in physical activity significantly decreased dementia risk (Hazard Ratio [HR]=0.60, p<.05), however, there was no significant relationship with smoking (HR=1.12, p=.58) and social contacts (1.06, p=.83). Our findings indicate physical activity is a promising modifiable lifestyle behavior for prevention. Future research should explore physical activity interventions that are most effective in reducing dementia risk, such as strength-based or aerobic-based activities.

